# mGluR1α expression in the hippocampus, subiculum, entorhinal cortex and superior temporal gyrus in Alzheimer’s disease

**DOI:** 10.1016/j.ibneur.2022.06.004

**Published:** 2022-06-22

**Authors:** J.H.Y. Yeung, T.H. Palpagama, C. Turner, H.J. Waldvogel, R.L.M. Faull, A. Kwakowsky

**Affiliations:** a Centre for Brain Research, Department of Anatomy and Medical Imaging, Faculty of Medical and Health Sciences, University of Auckland, Auckland, New Zealand; b Department of Anatomical Pathology, LabPlus, Auckland City Hospital, Auckland, New Zealand; c Pharmacology and Therapeutics, School of Medicine, Galway Neuroscience Centre, National University of Ireland, Galway, Ireland

**Keywords:** Glutamate receptor, MGluR1α, Hippocampus, Subiculum, Entorhinal cortex, Superior temporal gyrus, Alzheimer’s disease

## Abstract

Glutamate is the main excitatory neurotransmitter in the central nervous system, responsible for a plethora of cellular processes including memory formation and higher cerebral function and has been implicated in various neurological disease states. Alzheimer’s disease (AD) is the leading neurodegenerative disorder worldwide and is characterized by significant cell loss and glutamatergic dysfunction. While there has been a focus on ionotropic glutamatergic receptors few studies have attempted to elucidate the pathological changes of metabotropic glutamate receptors (mGluRs) in AD. mGluRs are G-protein coupled receptors which have a wide-ranging functionality, including the regulation of neuronal injury and survival. In particular, the group I mGluRs (mGluR1 and mGluR5) are associated with ionotropic receptor activation and upregulation with resultant glutamate release in normal neuronal functioning. The mGluR subtype 1 splice variant a (mGluR1α) is the longest variant of the mGluR1 receptor, is localized to dendritic processes and is mainly plasma membrane-bound. Activation of mGluR1a has been shown to result in increased constitutive activity of ionotropic receptors, although its role in neurodegenerative and other neurological diseases is controversial, with some animal studies demonstrating potential neuroprotective properties in excito- and neurotoxic environments. In this study, the expression of mGluR1a within normal and AD human hippocampal tissue was quantified using immunohistochemistry. We found a significantly reduced expression of mGluR1α within the stratum pyramidale and radiatum of the CA1subregion, subiculum, and entorhinal cortex. This downregulation could result in potential dysregulation of the glutamatergic system with consequences on AD progression by promoting excitotoxicity, but alternatively may also be a neuroprotective mechanism to prevent mGluR1α associated excitotoxic effects. In summary, more research is required to understand the role and possible consequences of mGluR1α downregulation in the human AD hippocampus, subiculum and entorhinal cortex and its potential as a therapeutic target.

## Introduction

Alzheimer’s Disease (AD) is one of the leading neurodegenerative disorders worldwide, with minimal effective therapeutics available for its management. Glutamate is the main excitatory neurotransmitter in the central nervous system (CNS), and dysfunction of the glutamatergic system has been implicated in many neurological disorders. Glutamate mediates its effects through receptors primarily categorized into two types – ionotropic and metabotropic. Ionotropic glutamatergic receptors are directly responsible for the influx and efflux of ions, and therefore have a direct rapid effect on the neuronal transmission (Yeung et al., 2021, Kew and John, 2005). Metabotropic glutamate receptors differ from ionotropic receptors in that they mediate slower responses through functional coupling with intracellular second messengers (Nakanishi, 1994). mGluRs have been implicated in the modulatory functions of various ion channels and GABAergic neurons (Conn and Pin, 1997, Nakanishi, 1994), the regulation of learning and memory (Menard and Quirion, 2012, Nakanishi, 1994, Zheng and Gallagher, 1992, Otani et al., 1993), and in the proper coordination and control of complicated motor tasks (Pekhletski et al., 1996).

There are at least 8 different subtypes of mGluR receptors, termed mGluR1 to mGluR8 (Tanabe et al., 1992, Abe et al., 1992, Okamoto et al., 1994, Saugstad et al., 1994). Each subtype displays regional selectivity and differential expression patterns (Tanabe et al., 1992). Categorization of the mGluRs are similar to that of the ionotropic receptors, in that they are separated based on their affinity to specific agonists. mGluRs are categorized into three groups; Group I (mGluR1/5) receptors are coupled to Gαq proteins and activate phospholipase C (PLC)-mediated hydrolysis of phosphoinositides to produce 1,4,5-triphosphate (IP3) to mobilize the release of intracellular Ca^2+^, Group II (mGluR2/3) and Group III (mGluR4/6/7/8) receptors are coupled to Gαi/o proteins and their activation inhibits adenylyl cyclase and cAMP formation, thereby limiting downstream protein kinase A (PKA) activation (Nakanishi, 1994).

The long variant, mGluR1a, is characterized by a unique sequence of 313 amino acids while the short variant, mGluR1b, has only 20 exclusive residues. The mGluR1a is unique among the different splice variants in that its longer C-tail results in the presence of unique protein binding domains (Ango et al., 2001, M Ribeiro, et al., 2010). Although the mGluR1/5 are often thought of as having similar functionality due to their classification and similarities in activation, the mGluR1α receptor appears to have functions distinctly separate from other Group I mGluRs (Valenti et al., 2002). mGluR1α has been implicated in the regulation of synapse plasticity, shown to induce increased dendritic spine density within the rat nucleus accumbens, with mGluR1α demonstrating opposing effects to mGluR5 indicating that although Group I mGluRs may have similarities in initial signaling transduction, subsequent effects and functionality can be highly diverse (Gross et al., 2016, Mannaioni et al., 2001).

mGluR1a has been shown to form complexes with other type I mGluRs within the hippocampus (Pandya et al., 2016). There is also further evidence of close interactions with other GPCRs such as adenosine, dopamine, and muscarinic receptors, with implications on the regulation of neurotransmitter release (Moldrich and Philip, 2003). As such, the dysregulation of its expression could have a significant impact on the wider functioning of the glutamatergic system.

Dysfunction particularly of Group I mGluRs have been implicated in AD neurotoxicity, while upregulation of Group II and III mGluRs have been shown to be neuroprotective in some studies (Nicoletti et al., 2011, Wroblewska et al., 1997, Bruno et al., 1997). This may be due to the inherent excitatory effect of Group I mGluR activation in contrast to the inherent inhibitory effect of Group II and III mGluR activation (Niswender and Conn, 2010). Gene knock-out studies have shown their importance in the normal regulatory function of the CNS, while Group II mGluR antagonist Ly341495 has demonstrated a neuroprotective effect on neurons, either by reducing tau phosphorylation or by enhancing neurogenesis, associated with improved learning in control mice (Higgins et al., 2004, Bruno et al., 2001). mGluRs may also play a role in regulating glutamate levels, as they are expressed within the postsynaptic and perisynaptic areas where they can readily detect any leakage of glutamate into the extrasynaptic space, with receptor activation correlating with the amount of glutamate released (Luján et al., 1997, Srivastava et al., 2020). Glutamate uptake is also regulated by astrocytic mGluRs, with Group II mGluRs minimizing glutamate spill-over when glutamate transporters are compromized (Wild et al., 2015). More recent studies have demonstrated direct interaction of mGluR1s with amyloid fibrils, with aberrant activation of receptors facilitating phagocytosis of hippocampal glutamatergic synapses (Wu et al., 2020). The presence of amyloid plaques also appears to trigger pyramidal cell hyperactivity through mGluR1 receptor activation (Ovsepian et al., 2017). Despite this, the role of mGluR1s in excitotoxicity remains a contentious topic. Whilst activation of mGluR1 has previously been shown to prevent NMDA-induced excitotoxicity in mouse hippocampal slice cultures, various studies have demonstrated synergistic effects with NMDARs (Baskys et al., 2005, Calo et al., 2005). It is therefore important to quantify expression alterations of mGluR1α in the human AD brain, as changes may infer alterations in mGluR function and may offer an explanation for other associated glutamatergic changes seen in the disease.

## Methods

### Human brain tissue preparation and neuropathological analysis

Donated post-mortem human brain tissue was obtained from the Neurological Foundation Human Brain Bank. The tissue was acquired through a donor program and the procedures were approved by the University of Auckland Human Participant’s Ethics Committee (Approval number: 011654). Processing of tissue followed the procedure described in Waldvogel et al. (2006). The right hemisphere of the brain was fixed by perfusion with 15 % formalin, cut into anatomical blocks, cryoprotected with sucrose solutions, and frozen at − 80 °C. Hippocampal (also containing the subiculum and entorhinal cortex) and STG blocks were used for this study. Nine control (Table 1) and eight AD cases (Table 2), with an average age of 78.5 years and maximum post-mortem time of 48 h were used for immunohistochemistry (IHC) experiments.

**Table 1:** Normal Human Brain Case Details Used for Immunohistochemistry.

Case	Age	Sex	PMD	Cause of Death	Weight (g)
H122	72	F	9	Emphysema	1230
H123	78	M	7.5	Aortic aneurysm	1260
H169	81	M	24	Asphyxia	1225
H180	73	M	33	Ischemic heart disease	1318
H181	78	F	20	Aortic aneurysm	1292
H202	83	M	14	Aortic aneurysm	1245
H226^a^	73	F	48	Mesothelioma	1279
H239^a^ H245	64 63	M M	15.5 20	Ischaemic Heart Disease Asphyxia	1529 1194

a Cases used for 3,3’-diaminobenzidine-peroxidase immunohistochemistry. Post-mortem delay (PMD).

**Table 2:** Alzheimer’s Disease Human Brain Case Details Used for Immunohistochemistry.

Case	Age	Sex	PM Delay	Cause of Death	CERAD Classification	Braak and Braak Score	Weight (g)
AZ45	82	M	4.5	Pneumonia	Probable AD	IV	1230
AZ88^a^	83	M	21	Pneumonia	Definite AD	IV	1121
AZ90	73	M	4	Gastrointestinal haemorrhage	Definite AD	IV	1260
AZ92	93	F	11.5	Bronchopneumonia	Probable AD	IV	1225
AZ98	91	F	20.5	Alzheimer’s dementia/atrial fibrillation	Definite AD	VI	1318
AZ102	84	F	14.5	Lower respiratory tract infection & hyaline arteriosclerosis	Definite AD	VI	1292
AZ103	87	M	< 24	Cerebrovascular accident	Definite AD	VI	1245
AZ113^a^	77	M	3.5	Alzheimer’s dementia/pneumonia	Definite AD	IV	1261

a Cases used for 3,3’-diaminobenzidine-peroxidase immunohistochemistry.

All control cases included in this study had no history of any primary neurodegenerative, psychiatric disorder, and neurological disease abnormalities, whereas all of the Alzheimer’s cases had clinical dementia. Standard sections, including the middle frontal gyrus, middle temporal gyrus, cingulate gyrus, hippocampus, caudate nucleus, substantia nigra, locus coeruleus, and cerebellum were examined from both control and AD groups by a neuropathologist. The distribution and density of tau and Aβ pathology were assessed immunohistochemically. The neuritic plaque density in the AD cases was classified into sparse, moderate, or frequent according to the criteria from the Consortium to Establish a Registry for Alzheimer’s disease (Mirra et al., 1991). Only those cases that fit this criterion for definite or probable AD were included in this study.

### Immunohistochemistry

Coronal sections of the hippocampus, subiculum, entorhinal cortex, and STG were cut on a freezing microtome at 60 µm and stored at 4 °C in phosphate-buffered saline (PBS) containing 0.1 % sodium azide. Two hippocampal and two STG sections, starting from the midpoint of the anterior commissure + 21.2 mm for the hippocampal block (containing the hippocampus, subiculum, and entorhinal cortex, plate 38–41) and + 9.3 mm for the STG block (plate 29–33 according to the Mai, Paxinos and Voss brain atlas (Mai et al., 2008), for each control and AD case, were immunostained with mGluR1α and NeuN specific antibodies. Free-floating 3,3’-diaminobenzidine (DAB)-peroxidase and fluorescent IHC were utilized for the visualization of mGluR1a, using a method published previously (Kwakowsky, Calvo-Flores Guzman et al., 2018; Waldvogel et al., 2006). All antibody dilutions were optimized. Primary antibodies and dilutions are described in Table 3. The omission of the primary antibodies resulted in a complete absence of immunoreactivity. Antibodies against mGluR1a and NeuN were diluted in 1 % normal donkey serum, and 0.04 % merthiolate in PBS (immunobuffer).

**Table 3:** Primary antibodies used in this study.

Antigen	Immunogen	Source, Host, Species, Catalogue Number	Dilutions
mGluR1a	Chinese hamster ovary cell line CHO-derived recombinant human mGluR1a Ser33-Ser522	R&D, Sheep, AF4836, RRID:AB_xxxxxx	1:500-fIHC 1:1000-DAB IHC
Anti-Neuronal Nuclei (NeuN)	Purified cell nuclei from mouse brain	Millipore, Rabbit, ABN78, RRID:AB_10807945	1:1000

### DAB-peroxidase immunohistochemistry

DAB-peroxidase IHC was performed as described by Kwakowsky et al. (2018). In brief, sections were washed in PBS with 0.2 % Triton X-100 (PBST) before blocking for endogenous peroxidases (50 % methanol and 1 % H_2_O_2_) for 20 min, followed by three 10-minute washes in PBST and incubated for 72 h in mGluR1a primary antibody in immunobuffer at 4 °C (Table 3). The sections were then washed in PBST before incubation for 24 h with the biotinylated secondary antibody (anti-sheep IgG-Biotin antibody produced in donkey, 1:1000, B7390, Sigma, St. Louis, MO, USA) in immunobuffer at room temperature (RT). The sections were then washed in PBST before incubation with ExtrAvidin (1:1000, E2886; Sigma, St. Louis, MO, USA) in immunobuffer for 4 h at RT, followed by three 10-minute washes in PBST before development in 0.05 % DAB and 0.01 % H_2_O_2_ in 0.1 M phosphate buffer. Sections were washed in PBST and mounted onto glass slides, dried, dehydrated through a graded series of ethanol, and cleared in xylene. The slides were coverslipped with DPX mountant (1019790500; Merck, Whitehouse Station, NJ, USA). The sections were imaged on either a Leica DMRB light microscope or a Leica MZ6 microscope (Wetzlar, Germany).

### Fluorescent immunohistochemistry

A total of 13 cases, 7 control and 6 CE, were used in this experiment. However, additional cases were included to account for potential problems such as tissue damage. Free-floating fluorescent IHC was performed as described previously by Kwakowsky et al. (2018). In brief, sections were incubated in PBST overnight at 4 °C followed by three 10-minute washes with PBST and incubation for 72 h in the primary antibodies mGluR1a and NeuN diluted in immunobuffer at 4 °C (Table 3). Sections were washed three times for 10 min in PBST before addition of secondary antibodies donkey anti-sheep Alexa Fluor 647 (1: 500, A21448, RRID:AB_2535865; Invitrogen), donkey anti-rabbit Alexa Fluor 488 (1: 500, A11034, RRID:AB_2576217; Invitrogen), and incubated for a further 24 h at RT. Sections were then washed for 10 min in PBST before incubation for 35 min at RT with Hoechst nuclei counterstain (1:10,000, 33342, RRID:AB_10626776, Invitrogen) diluted in PBS. After three subsequent 10-minute washes in PBS, sections were mounted onto glass slides, coverslipped with Mowiol mounting medium, and sealed with nail varnish.

### Imaging and analysis

Imaging was conducted using a Zeiss 710 inverted confocal laser-scanning microscope (Carl Zeiss, Jena, Germany). Brain regions and layers were differentiated based on cell type and relative location, utilizing NeuN and Hoechst staining. An argon laser was used to excite NeuN-positive neurons at a 488-nm wavelength, a helium-neon laser with a 633 nm wavelength was used for Alexa 647 immunolabeled antigens of interest, and a blue diode laser with a 405 nm wavelength was used for Alexa 405 for Hoechst counterstained nuclei with a 20x objective. Using ImageJ software (U. S. National Institutes of Health, Bethesda, Maryland, USA), after background subtraction and grayscale threshold determination, the glutamate receptor subunit density measurements were performed from a 31,000 µm^2^ area in each analyzed layer in the dentate gyrus (str. granulosum, str. moleculare and hilus), CA1, CA2, and CA3 (str. oriens, str. pyramidale, str. radiatum). Density measurements for the subsequent regions were measured using the following parameters: a 432,000 µm^2^ region in the subiculum, a 605,000 µm^2^ region in the entorhinal cortex, and a 692,000 µm^2^ region in the STG through all cortical layers. Both the threshold and the size of the region of interest were constant across all sections for each region in each experiment. The analysis was performed blinded to the experimental groupings to eliminate bias during the experiment, including image acquisition and analysis. Two hippocampal and two STG tissue sections from each case were randomized following standard simple randomization procedures in a blinded fashion.

### Statistical analysis

To examine differences between groups, an unpaired Mann Whitney test was used as the data did not meet the assumptions of parametric tests assessed by the D'Agostino–Pearson omnibus and Brown–Forsythe tests. No data points were identified and excluded as outliers using the ROUT method. All statistical analyses were conducted using Graph-Pad Prism software version 8 (GraphPad software; RRID:SCR_002798) with a value of p ≤ 0.05 considered significant. Adobe Photoshop CC 2018 (Adobe Systems Software, San Jose, CA, USA) was used to prepare the figures. All experimental data are expressed as the mean ± Standard Error of Mean (SEM).

## Results

**Fig. 1:**
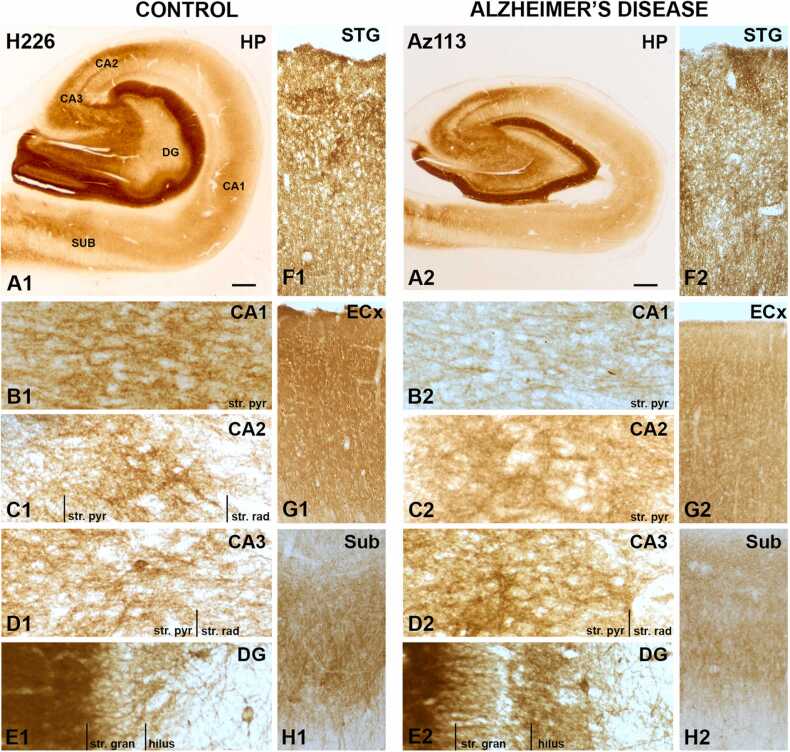
mGluR1α expression in the human hippocampus, subiculum, entorhinal cortex and superior temporal gyrus in control and AD cases visualized by 3,3’-diaminobenzidine-peroxidase immunohistochemistry. *A1-H1, A2-H2.* mGluR1α was particularly strong within the stratum (str.) moleculare of the dentate gyrus. AD cases displayed decreased immunoreactivity particularly within the CA1 region, subiculum and entorhinal cortex. CA = cornu ammonis; DG = dentate gyrus; ECx = entorhinal cortex; HP = hippocampus; STG = superior temporal gyrus; str. pyr = stratum pyramidale; str. rad = stratum radiatum; str. gran = stratum granulosum; Sub = subiculum. Scale bars: A1–2 = 1000 µm; B1-E1, B2-E2 = 100 µm; F1-H1, F2-H2 = 400 µm.

mGluR1α DAB immunoreactivity displayed strong diffuse staining within the CA1, 2 and 3 regions, mainly labeling surrounding neuronal cell bodies and associated processes (Fig. 1, A1). The str. molecular of the DG was particularly strongly stained, which appeared preserved in AD. However, AD cases exhibited decreased expression within the CA1, subiculum and entorhinal cortex (Fig. 1, B2, G2, H2).

**Fig. 2:**
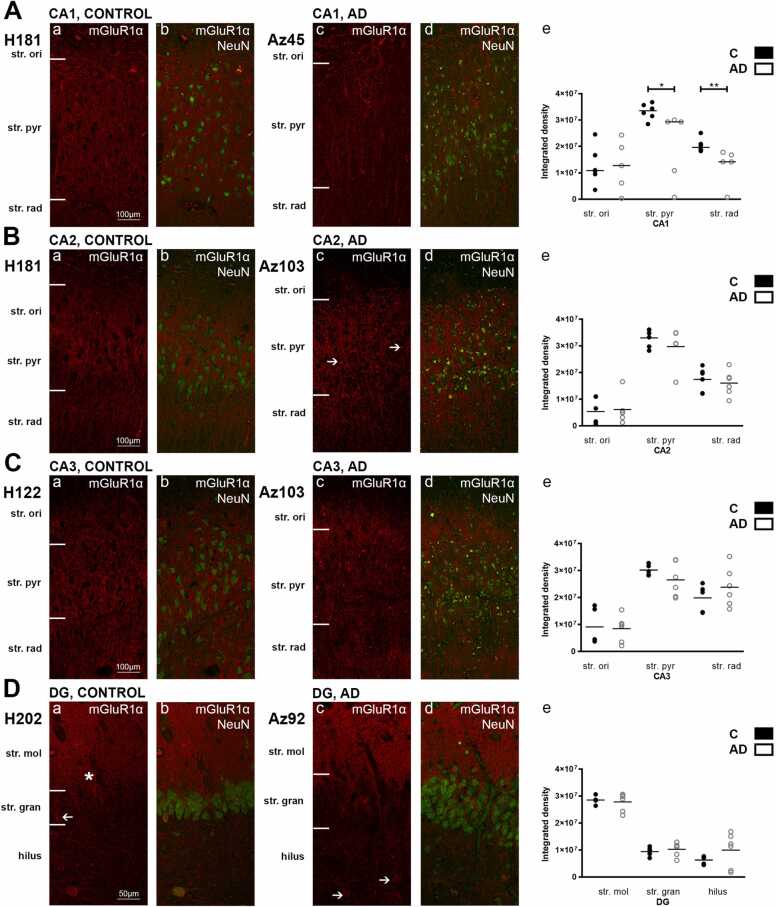
mGluR1α expression and quantification of immunoreactivity in the hippocampus in human control and Alzheimer’s disease cases visualized by fluorescent immunohistochemistry. Photomicrographs of representative regions of the CA1(A), CA2 (B), CA3 (C), and dentate gyrus (D) showing mGluR1α (red) and mGluR1α overlaid with NeuN (green) immunoreactivity for representative Alzheimer’s disease and control cases. In the hippocampal CA2, CA3, and DG subfields, mGluR1α density shows no statistically significant change in AD (white bars; n = 6) compared to control (black bars; n = 7) cases (Unpaired Mann-Whitney test). The figure shows a significant decrease in mGluR1α expression in the stratum (str.) pyramidale (*p ≤ 0.05), and radiatum (**p ≤ 0.01) of the CA1 region in AD cases. Data is expressed as mean with individual data points representing single cases. AD = Alzheimer’s disease; CA = cornu ammonis; DG = dentate gyrus; str. ori = stratum oriens; str. pyr = stratum pyramidale; str. rad = stratum radiatum; str. mol = stratum moleculare; str. gran = stratum granulosum. Scale bars A-C= 100 µm; D = 50 µm. (For interpretation of the references to colour in this figure legend, the reader is referred to the web version of this article.)

In fluorescence, the str. pyramidale in the hippocampal CA1, CA2, and CA3 regions displayed strong mGluR1α immunoreactivity in both control and AD cases (Fig. 2, A-C). mGluR1α labeling was observed along dendritic processes, with less staining around neuronal cell bodies. The str. oriens revealed weaker immunoreactivity, with labelling primarily seen along dendritic and axonal processes. The CA1 region in AD displayed much weaker immunoreactivity within the str. pyramidale and str. radiatum with significantly reduced diffuse staining, although strong immunolabelling along neuronal fibres was maintained. mGluR1α immunoreactivity along neuronal processes within the str. oriens remained unchanged in AD compared to control brains (Fig. 2A, c).

mGluR1α immunoreactivity within the CA2 followed a similar pattern to that of the CA1. Labeling appeared concentrated around neuronal bodies of the str. pyramidale, with processes extending into the str. radiatum and the str. oriens (Fig. 2B, a). In AD, immunoreactivity within the CA2 remained similar compared to controls, with an increase neuronal localisation, with labeling of neuronal cells within the str. pyramidale (Fig. 2A, c, arrows). Labeling patterns within the CA3 followed that seen within the CA2 region.

The DG displayed very dense immunoreactivity within the str. moleculare, both in control and AD cases. Occasional immunolabeling along neuronal fibres can be seen, although the majority of labeling within the str. moleculare is diffuse. Labeling was seen along neuronal processes extending into the str. granulosum (Fig. 2D, a, asterisk). Immunoreactivity within the str. granulosum revealed weaker staining, with occasional localisation to neuronal cell bodies (Fig. 2D, a, arrow). The hilus displayed similar immunoreactivity to the str. granulosum, with some labeling within cell bodies and also surrounding neuronal bodies, particularly within the AD cases (Fig. 2D, c, arrows).

**Fig. 3:**
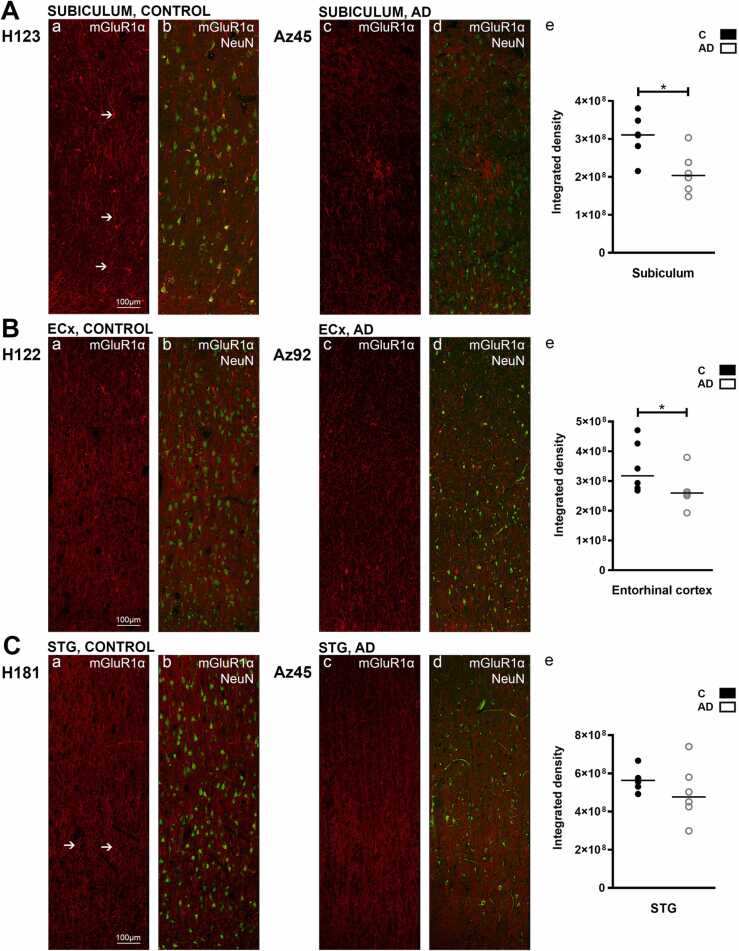
mGluR1α expression and quantification of immunoreactivity in the subiculum, entorhinal cortex and superior temporal gyrus in human control and Alzheimer’s disease cases. Photomicrographs of representative regions of the subiculum (A), entorhinal cortex (B), and superior temporal gyrus (C) showing mGluR1α (red) and mGluR1α overlaid with NeuN (green) immunoreactivity for representative Alzheimer’s disease and control cases. In the STG, mGluR1α density shows no statistically significant change in AD (white bars; n = 6) compared to control (black bars; n = 7) cases (Unpaired Mann-Whitney test). The figure shows a significant decrease in mGluR1α expression in the subiculum (*p ≤ 0.05) and entorhinal cortex (*p ≤ 0.05) in AD cases. Data is expressed as mean with individual data points representing single cases. AD = Alzheimer’s disease; ECx = entorhinal cortex: STG = superior temporal gyrus. Scale bars A-C = 100 µm. (For interpretation of the references to colour in this figure legend, the reader is referred to the web version of this article.)

The subiculum and the entorhinal cortex displayed strong mGluR1α immunoreactivity. The subiculum revealed strong labeling around cell bodies and adjacent processes in control cases (Fig. 3A, a, arrow). Extensive fibrous expression can be seen throughout the subiculum, possibly labeling cellular processes, but occasional labeling of neurons could also be seen. Expression within AD cases was significantly reduced, whilst fibrous labeling remained preserved, with reduced cellular and neuronal labeling (Fig. 3A, c). Entorhinal mGluR1α immunoreactivity exhibited similar labeling patterns as in the subiculum, with AD cases displaying markedly reduced immunolabeling (Fig. 3B, e). mGluR1α labeling within the STG exhibited similar patterns to the entorhinal cortex and subiculum, along the processes and around cell bodies (Fig. 3C, a, arrow). In the CA1 region, the str. pyramidale (p ≤ 0.05) and str. radiatum (p ≤ 0.01) displayed a statistically significant decrease in mGluR1α expression, whilst the str. oriens immunoreactivity remained unchanged (Fig. 2A, e). Within the subiculum and entorhinal cortex, expression was significantly (p ≤ 0.05) lower in AD cases compared to control (Fig. 3A, e; 3B, e).

## Discussion

We report decreased mGluR1α expression within the str. pyramidale and radiatum of the CA1, subiculum, and entorhinal cortex in AD compared to control in human cases. Our study is the first to examine mGluR1α expression within the AD human hippocampus, subiculum, entorhinal cortex and STG and compare the receptor density with healthy controls. The distinction of mGluR1α is vital from generalized mGluR1 expression due to its specific postulated roles in neuroprotective and excitotoxic disease processes. Our results indicate a region-specific downregulation of mGluR1α expression within the str. pyramidale and radiatum of the CA1, the subiculum, and entorhinal cortex.

Western blot analysis of Group I mGluRs within the frontal cortex in Lewy Body Dementia and AD has demonstrated a decrease in expression of mGluR1 correlating with AD severity, but no significant changes in mGluR5 (Albasanz et al., 2005). Correlation of mGluR1 expression with severity hints at a strong association with neuropathological changes. An autoradiography study measuring ^3^H-glutamate binding at mGluR sites display decreased binding within the subiculum and the CA1 hippocampal region in AD (Dewar et al., 1991). A previous PET study examining mGluR1 availability within the cerebellum and frontal, parietal, and temporal cortices in early AD yielded no significant changes, however the hippocampus, which is one of the earliest brain regions to be affected, was not examined (Ishibashi et al., 2019). Interestingly, animal studies, primarily transgenic APPswe/PS1dE9 and Aβ_1–40_-injection models, have demonstrated an acute increase in mGluR1 expression (Bie et al., 2019, Ostapchenko et al., 2013). Whether this indicates an acute reaction not noted in human studies, or whether it is because these mouse models do not fully replicate the human disease process, will require further investigation.

Previous studies have demonstrated mGluR1α expression localized to the cell bodies of CA1 pyramidal neurons (Ong et al., 1998). mGluR1α expression within dendritic processes has been reported, with dendrites and dendritic spines displaying positive double-labeling for mGluR1α and ionotropic GluR2/3 using immunocytochemistry and electron microscopy of rat hippocampi (Ong et al., 1998). Studies using immunohistochemical and in situ hybridization have observed mGluR1α expression within GABAergic neurons in the rat, which builds on its role as a regulatory modulator within the CNS (Shigemoto et al., 1992). Its activation may cause the inhibition of GABAergic neurons, as mGluR1 activation within rat CA1 pyramidal cells leads to somatic calcium transients and neuronal depolarization (Mannaioni et al., 2001). In the AD brain, the reduced mGluR1α expression might help to dampen this process and reduce excitotoxicity. The complex remodeling of the GABA_A_ receptor subunit composition and changes in expression levels might also help to compensate and/or maintain the fine balance between excitation and inhibition, but the order of events and the interplay between these receptors has to be further investigated (Kwakowsky et al., 2018).

mGluR1α expression is localized within the perisynaptic regions of the neuron (Lujan et al., 1996). The localization of mGluR1α to this area places it in a unique position, ideal for the regulation of glutamate, as glutamate would not diffuse to the perisynaptic region unless excessive glutamate release or altered glutamate reuptake occurs. There is evidence of Group I mGluR-mediated AMPA endocytosis, which may be a neuroprotective mechanism during aberrant glutamate release (Casimiro et al., 2011). mGluR1α activation has been demonstrated to protect against NMDAR excitotoxicity, with a study demonstrating a Group I mGluR-mediated reduction in NMDA currents in rat hippocampal neurons (Blaabjerg et al., 2003). In addition, the application of mGluR1 selective antagonist LY367385 abolished this neuroprotective effect, whilst mGluR5 antagonism had no significant effect. As such, a decrease in mGluR1α expression, particularly within vulnerable regions such as the CA1 and subiculum, could exacerbate NMDAR excitotoxicity. mGluR1α is required for the induction of long-term potentiation (LTP) in certain hippocampal inhibitory neurons (Perez et al., 2001). In certain brain regions, LTP formation within somatostatin interneurons has been shown to be fully reliant on mGluR1α activity, with transgenic mGluR1α knockout mice demonstrating an absence of LTP induction within interneurons of the hippocampal str. radiatum (Honoré et al., 2021, Lapointe et al., 2004). Therefore, mGluR1α downregulation could also impair cognitive function and memory formation, through both compromized LTP production, and impaired activation of inhibitory interneurons, further exacerbating potential excitotoxic damage.

Conversely, some studies suggest an upregulation in mGluR1α activity is responsible for the excitotoxic damage observed in AD. Under conditions of excitotoxicity, Group I mGluRs can be activated for extended periods of time, leading to massive increases in postsynaptic and cytosolic calcium. The activation of mGluR1α has been shown to lead to rapid production of IP3, causing downstream activation of intracellular Ca^2+^ release (Fagni et al., 2000). Likewise, injection of Group I mGluR agonist (S)− 3,5-dihydroxyphenylglycine (DHPG) intracerebroventricularly results in ionotropic glutamate receptor alterations in the hippocampus and a significant loss of pyramidal neurons within 4–7 days (Ong and Balcar, 1997). Recently, administration of novel therapeutic JBPOS0101, a mGluR1 and 7 antagonist, into a 5XFAD AD mouse model demonstrated relative attenuation of memory loss and reduction in amyloid precursor protein and Aβ_1–42_, with authors postulating its effects being due to antagonism of primarily mGluR7, but also mild effects on mGluR1 and 5 (Park et al., 2019).

The question remains as to how mGluR1α has been neuroprotective in some studies, but excitotoxic in others. Xu et al. (2007) demonstrated a novel mechanism through which the truncation of mGluR1α may lead to its excitotoxic effect, while normal physiological function would confer neuroprotective properties. Xu et al. (2007) discovered a calpain-mediated truncation of the C-terminal domain in mGluR1α which annulled the Homer-PI3K-Akt pathway involved in preventing neuronal apoptosis (Rong et al., 2003). In addition to this, Xu et al. (2008) discovered that NMDAR activation plays a significant role in triggering calpain-mediated truncation. Truncation of mGluR1α, therefore, leads to a transition from a neuroprotective to a neurodegenerative state. The prevention of this truncation has been shown to be neuroprotective against excitotoxicity in rat neuronal cultures (Xu et al., 2008). In addition, the application of protein Homer 1a is able to ameliorate neuronal damage in artificial TBI through modulation of group I mGluRs (Luo et al., 2014). Glutamate excitotoxicity is a well-recognised complication of TBI. Such phenomenon may result in mGluR truncation as described by Xu et al. (2008), resulting in mGluR transitioning from a neuroprotective to excitotoxic activity, and may explain why Homer1a reduced neuronal injury through disruption of mGluR1. This significant finding may explain why some studies describe mGluR1α as a neuroprotective receptor, while others observed neurotoxicity with mGluR1α stimulation. This discovery complicates the viability of mGluR1α as a therapeutic target, particularly through classical antagonist/agonist avenues. Future directions may include the targeting of aberrant mGluR1α modifications to maintain and preserve normal physiological receptor functions.

Another factor for consideration in future studies is the comparison between female and male cases. There has been documentation of metabolic interactions between estrogen receptors and mGluRs, wherein female rodents demonstrate estrogen receptor-dependent recruitment of mGluRs not observed in male rodents, with implications on physiological responses to drug addiction (Eisinger et al., 2018). It is yet to be seen whether the difference in estrogen exposure over a human lifespan has any effect on the later stages of life, particularly given estrogen levels are significantly reduced in a female’s later years, with an estimated estrogen level loss of 80 % in their first year of menopause (Horstman et al., 2012). Our group has previously investigated sex-associated differences in GABA receptor subunit and transporter expression, which demonstrated some expression changes within the STG between males and females, and also noted different expression trajectories between young and old cases for specific GABA subunits that were different in male and female groups (Ethiraj et al., 2021, Pandya et al., 2019). As such, future consideration of the potential implications of sex on mGluR1α expression may demonstrate important distinctions in cellular processes, although the significance of these differences may be minimal in the particular cases examined in this study given the post-menopausal stage of all cases. Indeed, we did not observe notable differences in mGluR1 α expression between female and male cases in the currents study, but the number or cases is too low to draw any strong conclusions.

The GluN1 and GluN2A, subunits of the ionotropic NMDARs, are both upregulated within the AD hippocampus (Yeung et al., 2021, Kwakowsky et al., 2018). Ionotropic receptors have long been associated with excitotoxicity in AD progression, with interactions between NMDARs and histopathological hallmarks of AD resulting in the propagation and upregulation of NMDAR activity (Liu et al., 2019). Whilst metabotropic receptors play a role in modulating the responses of ionotropic receptors, whether the downregulation of mGluR1α expression we have observed in this study is an upstream or downstream effect of interactions between ionotropic receptors or pathophysiological processes is difficult to conclude. Given the potential neuroprotective effects of mGluR1 such as inducing AMPAR endocytosis and reducing NMDAR activity noted earlier in this discussion, its reduction in AD could potentially exacerbate glutamatergic excitotoxicity. The relationship between ionotropic and metabotropic receptors has not completely been elucidated, far less so in the context of disease and neurodegeneration. In addition, recent studies have demonstrated metabotropic effects of the NMDAR, long thought to be purely an ionotropic receptor (Chung, 2013). Understanding the complex interplay between these different types of glutamatergic and in addition the GABAergic receptors and their subunits should be regarded as a priority in future neurochemical research, and may offer potential exciting answers to the neurotransmitter and synapse dysregulation in AD and other neurodegenerative disorders.

In conclusion, the mGluR1α is a currently understudied receptor, which potentially plays a specific and direct role in modulating the AD disease process. Ours is the first study to quantify layer- and region-specific alterations in mGluR1α expression within the human AD hippocampus, subiculum, entorhinal cortex and STG. mGluR1α downregulation worsening with the progression of pathology in the frontal cortex and possibly in these structures of the medial temporal lobe implicate its dysfunction as a major contributor to AD. Further examination of other mGluRs in the human brain, particularly that of mGluR5, would offer increased insight into disease mechanisms. Encouragingly, there is increasing research examining the impact of modulating mGluR1α in animal models of neurological and neurodegenerative disease processes, which offers some insight into its potential therapeutic properties. Therefore, with more research aiming to understand the role and possible consequences of mGluR1α downregulation in the human AD brain, novel promising therapeutic targets could be identified.

## Funding

This work was supported by Alzheimers New Zealand Charitable Trust (AK; 370836), Alzheimers New Zealand (AK; 3718869), Freemasons New Zealand (AK; 3719321), Aotearoa Foundation, Centre for Brain Research and 10.13039/501100001537University of Auckland (AK; 3705579), 10.13039/501100001505Health Research Council of New Zealand (RF and HW; 3627373), 10.13039/501100001543Neurological Foundation of New Zealand (TP and AK; 3715525), and 10.13039/501100014191Brain Research New Zealand.

## Ethics statement

All procedures were approved by the University of Auckland Human Participants' Ethics Committee (Approval number: 001654).

## Author contribution

Y.J.H.Y., T.H.P., H.J.W., A.K. performed research; A.K., R.L.F. designed research; C.T. performed pathological assessment; Y.J.H.Y., R.L.F., A.K. wrote the paper.

## Competing financial interests

The authors declare no competing financial interests.

## Data Availability

The original contributions presented in the study are included in the article, further inquiries can be directed to the corresponding author.
